# Machine learning method for the classification of the state of living organisms’ oscillations

**DOI:** 10.3389/fbioe.2024.1348106

**Published:** 2024-03-07

**Authors:** David Kweku, Maria I. Villalba, Ronnie G. Willaert, Osvaldo M. Yantorno, Maria E. Vela, Anna K. Panorska, Sandor Kasas

**Affiliations:** ^1^ Department of Mathematics and Statistics, University of Nevada Reno, Reno, NV, United States; ^2^ Laboratory of Biological Electron Microscopy, Ecole Polytechnique Fédérale de Lausanne (EPFL) and University of Lausanne, Lausanne, Switzerland; ^3^ International Joint Research Group VUB-EPFL BioNanotechnology and NanoMedicine (NANO), Brussels, Switzerland; ^4^ Research Group Structural Biology Brussels, Alliance Research Group VUB-UGhent NanoMicrobiology (NAMI), Vrije Universiteit Brussel (VUB), Brussels, Belgium; ^5^ Centro de Investigación y Desarrollo en Fermentaciones Industriales (CINDEFI), Facultad de Ciencias Exactas, Universidad Nacional de La Plata—CONICET, Buenos Aires, Argentina; ^6^ Instituto de Investigaciones Fisicoquímicas Teóricas y Aplicadas (INIFTA), Universidad Nacional de La Plata—CONICET, Buenos Aires, Argentina; ^7^ Centre Universitaire Romand de Médecine Légale, Unité facultaire d’anatomie et de morphologie (UFAM), Université de Lausanne, Lausanne, Switzerland

**Keywords:** artificial intelligence, machine learning, atomic force microscopy, cellular nanomotion, bacterial virulence, *Bordetella* pertussis

## Abstract

The World Health Organization highlights the urgent need to address the global threat posed by antibiotic-resistant bacteria. Efficient and rapid detection of bacterial response to antibiotics and their virulence state is crucial for the effective treatment of bacterial infections. However, current methods for investigating bacterial antibiotic response and metabolic state are time-consuming and lack accuracy. To address these limitations, we propose a novel method for classifying bacterial virulence based on statistical analysis of nanomotion recordings. We demonstrated the method by classifying living *Bordetella pertussis* bacteria in the virulent or avirulence phase, and dead bacteria, based on their cellular nanomotion signal. Our method offers significant advantages over current approaches, as it is faster and more accurate. Additionally, its versatility allows for the analysis of cellular nanomotion in various applications beyond bacterial virulence classification.

## 1 Introduction

According to the World Health Organization (WHO), the spread of antibiotic-resistant bacteria is currently one of the biggest threats to global health, food security, and development (World Health Organization, 2023). Without action, even minor infections or injuries can become life-threatening, as was the case in the pre-antibiotic era a century ago. At present, several factors contribute to the spread of resistant bacterial strains, with the misuse and overuse of antibiotics playing a predominant role. The use of broad-spectrum antibiotics, which are effective against a wide variety of microorganisms, further accelerates the spread of antibiotic-resistant species. These types of drugs are routinely used in medical centers worldwide since antibiotic sensitivity tests, which determine the susceptibility of a bacterial strain to different antibiotics, can take anywhere from 24 h for common germs to several days for bacteria such as *Bordetella*, the causative agent of whooping cough (Li et al., 2019). Unfortunately, their use is necessary to initiate treatment for newly admitted patients with bacterial infections before the completion of sensitivity tests, which are based on sufficient cell growth of the pathogenic microorganism and, therefore, can take several days to week. To reduce the time delay between patient admission and identification of the most appropriate antibiotic, numerous options exist, but none have been widely implemented in hospitals thus far. One promising option for the rapid determination of bacterial sensitivity to antibiotics involves detecting the nanometric scale oscillations that characterize all living organisms (Kasas et al., 2015).

Detecting nanometer-scale oscillations in living organisms offers a promising method for determining cell viability. These low amplitude oscillations were initially detected using atomic force microscopy (AFM) (Binnig et al., 1986). The method involves attaching the organism of interest to a microfabricated cantilever and recording its oscillations over time in the presence of various chemicals in the analysis chamber (Longo et al., 2013). AFMs are extremely sensitive devices that can detect cantilever displacements with sub-Angstrom resolution. Consequently, they can monitor bacterial nanomotion that is 1–2 orders of magnitude larger than the instrument’s detection limit.

Numerous studies involving bacteria, yeast, plant, and mammalian cells have demonstrated that monitoring the nanomotion of living cells holds promise as a label-free method for assessing life-death transitions (Longo et al., 2013; Kasas et al., 2015; Pleskova et al., 2023a; b; Starodubtseva et al., 2023; Villalba et al., 2022). The exact origin of the nanomotion signal is still unclear. Various factors such as flagella, ion channel activity, or conformational changes in surface proteins can induce these small oscillations. Despite the mysterious nature of the nanomotion signal’s origin, several independent research teams have proposed promising applications based on nanomotion analysis.

One of the primary and most straightforward applications is the development of rapid and label-free antimicrobial sensitivity tests for pathogenic bacteria (or yeasts) (Stupar et al., 2017; Mustazzolu et al., 2019; Villalba et al., 2022; 2023; Radonicic et al., 2023). In this approach, the organism of interest is exposed to different antibiotics, and its nanomotion is monitored over time. Such a device allows for antibiotic sensitivity tests to be completed within 1–3 h (Villalba et al., 2018). Nanomotion analysis has also been proposed for monitoring cancer cell sensitivity to chemicals (Wu et al., 2016; Stupar et al., 2021) and as a chemistry-independent life detector for extraterrestrial life research (Lissandrello et al., 2014; Kasas et al., 2015).

Cellular nanomotion patterns not only reflect the life/death state of an organism but also provide insights into its metabolic activity level and, in some cases, bacterial virulence. It is known that *Bordetella pertussis,* the bacteria responsible for whooping cough can adopt different states of virulence, such as being avirulent or virulent. Each virulence phase involves the expression of specific genes. Virulence factor genes (such as adhesins and toxins) are expressed during a virulent state (Cotter and Jones, 2003). Meanwhile, avirulent metabolism may be involved in bacterial survival, transmission, and/or persistence (Moon et al., 2017). This bacterium exhibits distinct nanomotion patterns in its virulent and avirulent states. These patterns are characterized by variations in cantilever displacements. This observation strongly suggests that nanomotion signals carry significant information about the studied organism. So far, nanomotion data have been analyzed primarily through variance and displacement distribution histograms. However, to fully harness the information embedded in nanomotion signals and achieve precise classifications (such as distinguishing between life and death states), a more rigorous approach is required.

From a statistical perspective, the task of identifying the virulence state represented by a given nanomotion recording can be viewed as a clustering-classification problem. Clustering methods play a critical role in various domains, including data analysis, machine learning, and pattern recognition. These methods aim to group data points together based on their inherent characteristics or similarity measures. Traditional clustering algorithms, such as k-means and hierarchical clustering, are widely used (Ester et al., 1996; Hahsler et al., 2019; Hahsler and Piekenbrock, 2022). More advanced approaches include density-based clustering, model-based clustering (Hastie and Tibshirani, 1996; Fraley and Raftery, 2002; Scrucca et al., 2016), random forests (Breiman, 2001; Breiman et al., 2022), and spectral clustering (Ng et al., 2002). In our analysis, we employed Gaussian mixture models, which are commonly used and numerically implemented in various software packages (Fraley and Raftery, 2002; Scrucca et al., 2016; Scrucca, 2022). These clustering methods offer valuable tools for grouping similar nanomotion recordings and aiding in the classification of virulence states.

In this work, we introduce a novel artificial intelligence (AI)-based method for analyzing cellular nanomotion signals. This AI approach is a versatile technique applicable to the analysis of nanomotion observations across various organisms. To illustrate the method, we present its application on analyzing living *B. pertussis* in two different virulence phases, and dead bacteria.

## 2 Materials and methods

### 2.1 Bacteria culture preparation and conditions

In this study, two strains of *B. pertussis* were selected: an avirulent phase-locked mutant *B. pertussis* 537 (Relman et al., 1990), and the reference strain *B. pertussis* Tohama I. The bacteria were cultured from frozen stocks on *Bordetella* agar plates supplemented with 15% (v/v) horse blood (BD Difco). Colonies were incubated for 72 h at 37°C and subsequently regrown for 48 h on a fresh agar plate under the same conditions.

To initiate the liquid culture, an agar plate was inoculated into a 30 mL volume of Stainer-Sholte (SS) liquid medium (Stainer and Scholte, 1970) in a 50 mL flask. The flask was incubated at 160 rpm and 37°C for 24 h. The bacterial liquid culture was then subjected to centrifugation at 8500 rpm for 5 minutes and thoroughly washed three times with phosphate-buffered saline (PBS, pH 74) (Sigma-Aldrich). Finally, the pellet was resuspended in SS liquid medium to achieve a final concentration of 10^6^–10^8^ colony-forming units/ml (CFU/ml).

We determined if the culture is viable or not by measuring the optical density (OD) of the liquid culture after 24 h: OD will remain low (less than 0.2) if the cells do not grow; and will increase to at least 1–1.5 (at 650 nm) if the cells are in good shape and grow. Because PBS is documented as a harmless buffer, we did not consider measuring the viability of the cells between the washing and the cell adhesion steps.

Two different protocols have been used to verify the viability of the cells after the attachment procedure (including washing steps). The first consisted of staining the bacteria with live/dead stain (propidium iodure and syto9) and the second of applying mechanical force to detach the cells and place them on a fresh agar plate to assess their growth after 24 h at 37°C.

To evaluate the *B. pertussis* virulence phase cells were grown on Bordet-Gengou agar medium (Difco) plates containing 15% defibrinated sheep blood. Plates were incubated 48 h at 37°C. The colony hemolytic phenotype was associated to the release of adenylate cyclase-hemolysin toxin (ACT) by cells expressing the virulence phase. ACT is one of the principal virulence factor produced by B. pertussis (Carbonetti et al., 2005).

### 2.2 Cantilever functionalization

For the measurements, rectangular AFM cantilevers (model: SD-qp-CONT tip-less cantilevers, NanoandMore, GmbH, Germany) with a nominal resonance frequency of 32 kHz and a force constant of 0.1 N/m were selected. These cantilevers were held in place using silicon support chips (SD-Align, Nanosensors, NanoWorld AG).

To enable the attachment of cells, the cantilevers were functionalized with adhesive molecules. This involved covering the sensor with a drop of 0.1% (w/v) poly-L-lysine solution (Sigma-Aldrich) for a duration of 5 min. After that, the cantilevers were removed from the poly-L-lysine drop and allowed to air dry for a few minutes. Subsequently, they were incubated with the *B. pertussis* suspension for 40 min at 37°C. Prior to placing the cantilever with the adhered bacteria in the analysis chamber, three washes with PBS were performed to remove the loosely bound cells.

Cantilever functionalization is a very important and challenging step in the sample preparation protocol. The cross linking molecule has to be harmless to the microorganism of interest, relatively unspecific to permit the attachment of various bacterial species and strong enough to permit liquid exchange during measurements. In our case the cantilevers were covered with an aqueous polylysine solution that crosslinks the negative charges of the Si_3_N_4_ surface and the bacterial cell wall. Functionalization and attachment were checked by imaging the cantilever with an optical microscope before and after the measurement.

Functionalization protocols such as the one used in this study can be found in several published works that required the adhesion of bacteria to surfaces or cantilevers (Oh and Hinterdorfer, 2018; Lisandrello et al., 2014; Vadillo-Rodríguez et al., 2004; Villalba et al., 2022).

### 2.3 Data acquisition

The nanomotion measurements were performed using an AFM-based nanomotion device developed at the Laboratory for Physics of Living Matter at Ecole Polytechnique Federale de Lausanne (EPFL, Lausanne, Switzerland) (Venturelli et al., 2021). The analysis chamber of the AFM nanomotion device was filled with SS liquid medium at room temperature, which had been filtered previously using a 0.22 µm pore size filter. Data recording commenced after a few minutes of placing the cantilever on the detector to allow for stabilization of the liquid inside the analysis chamber.

For data acquisition, LabVIEW software from National Instruments (Venturelli et al., 2020) was utilized to configure the parameters and handle the data acquisition process. The deflection signal was recorded over a duration of 30 min, with a sampling frequency of 20 kHz.

### 2.4 Machine learning classification of the nanomotion

The methodology employed in this study involves machine learning (ML) and artificial intelligence (AI) techniques to classify nanomotion data. The general approach is to divide a longer observation into shorter strands and train a classification algorithm using various ML/AI methods on these strands. We used Model Based Clustering (MDA) (Fraley and Raftery, 2002; Scrucca et al., 2016; Scrucca, 2022) and Random Forest (RF) (Breiman, 2001; Breiman et al., 2022). The trained algorithm is then employed to classify new observations (Kweku, 2021).

To obtain a trained classification algorithm, several steps are followed. First, the data undergoes preprocessing to prepare it for analysis. Next, dimension reduction techniques are applied to reduce the complexity of the data while retaining essential information. We used Functional Principal Components (FPCA) method for dimension reduction. Finally, the classifier is trained using appropriate ML/AI algorithms. Once the classifier is trained, it can be applied to classify new observations. Our approach for classifying a new observation is novel and described in detail in the corresponding section below.

In the result section, we provide a more detailed description of the steps involved in the classification process. It should be noted that the method outlined below can be applied to nanomotion observations from multiple bacterial states, denoted as K states. To maintain clarity and establish a connection with the example of classifying *B. pertussis* nanomotion, we will present the method specifically for the case where K = 3. The three states will be referred to as Virulent (V), Avirulent (AV), and Dead (D).

### 2.5 R packages and functions used

In addition to basic R, specialized R packages were used for the functional data work (smoothing and FPCA), and clustering/discriminant analysis work. For smoothing and creating FPCs, R package “fda” was used. The functions used for data smoothing were: create. bspline.basis, fdPar, smooth. basis, and eval. fd. Selection of the smoothing parameter was done using a cross-validation approach. Function pca. fd was used to compute the FPCs. Package “mclust” was used for clustering, discriminant, and classification work with the MDA method. Function mclustBIC was used to determine the clusters for checking the internal consistency and external variability. Function MclustDA was used to train the classifier. Package randomForest was used for the RF classification and prediction method.

## 3 Results

**FIGURE 1:**
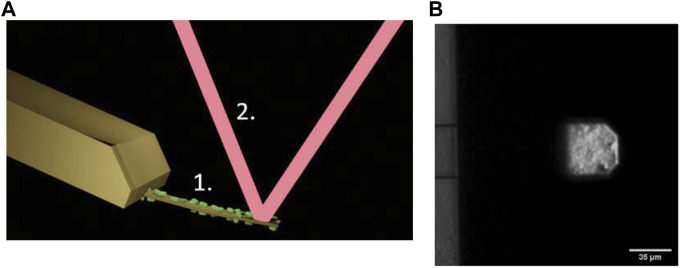
**(A)** Setup for AFM-based nanomotion detection: An I shaped cantilever (1) with living bacteria attached to its surface (in green). Bacterial nanomotion induces cantilever oscillations that are detected through the deflections of the laser beam (2) reflected off the end of the cantilever. **(B)** Optical microscope image of the cantilever holding chip (left) an I shaped cantilever (right) with B. pertussis attached to it. Only the very end of the lever is visible since this part is gold coated, the rest of the lever is transparent.

The setup of the AFM-based nanomotion detection and an image of the cantilever used in this work are presented in Figures 1A, B.

### 3.1 Preprocessing of the experimental data

The process commences with the partitioning of observations from all states (all experimental data) into shorter segments, termed as ‘strands’. We then randomly select strands from each state for the purpose of training the classification algorithm. These selected strands undergo scaling and smoothing to enhance the identification of trends. Subsequently, outlier strands are detected in each strand category and eliminated from the training set. Next, dimension reduction is done through the Functional Principal Components Analysis (FPCA). The FPCs scores will serve as data for all subsequent analyses. The final step involves assessing the strands from each state for internal consistency and external variability. For effective machine learning classification, it is crucial that data from the same state exhibits sufficient similarity (internal consistency), while data from different states demonstrates noticeable differences (external variability). More detailed explanations of the preprocessing steps follow.

Step 1: *Segmentation of Experimental Observations:* Assuming access to cantilever displacements (5-min or similar length observations of the displacement record - nanomotion) from all metabolic states of the target organism, it’s likely that these records will be extensive. As such, computational handling without high-powered computers may be challenging. Therefore, we recommend dividing these observations into shorter, more computationally manageable segments, referred to as chunks or strands. We divided 5 min long experimental observations into 5 s long chunks/strands.

Step 2: *Random Sampling for Classifier Training:* To enhance computational efficiency, a reasonable number of chunks are randomly selected from each state to be used for training the classifier. For our process, we utilized 30 training chunks from each state (total of 90 strands). These 90 strands were our initial *training set*. The following steps were performed on these training data.

Step 3: *Scaling of Training Strands:* For the purpose of classifying the bacterial state, our focus was the nanomotion pattern, rather than its magnitude. Hence, we advise scaling the training strands. This is achieved by dividing all displacements within each strand by their respective standard deviation (or range).

Step 4: *Smoothing Training Chunks:* Employ a functional data smoother, such as B-splines, to smooth the training chunks. This step aids in understanding the general pattern of the signal and reduces computational time for subsequent steps. Smoothing unveils common features and patterns in the data more effectively than raw displacement records.

Step 5: *Outlier Identification and Removal:* Outlier strands, which noticeably differ from the majority of the data, should be identified and removed. As the similarity of data within a state significantly influences classifier accuracy, the removal of outlier strands is recommended, since these can obscure the data pattern. Outliers can be detected by calculating the 25th and 75th quantiles of strand values. The Interquartile Range (IQR) is the difference between these percentiles. Any observation (strand) with a minimum value below the 25th percentile minus 1.5 times the IQR or a maximum value above the 75th percentile plus 1.5 times the IQR is considered an outlier. Outliers can arise upon external perturbations such as opening or closing a door, floating particles into the analysis chamber that crosses the laser beam during the measurement or any electrical or mechanical disturbance.

Step 6: *Dimension reduction.* In the dimension reduction step, the remaining training strands are subjected to FPCA to reduce their dimensionality. FPCA is used to capture the main sources of variation in the data by representing the observations in terms of functional principal components (FPCs). These FPC’s scores are then used as the data in place of the original displacement training set. Several FPCs which explain a good portion of the total variability (typically 85%–99%) in the training data are used for further analysis.

Step 7: *Checking internal consistency and external variability.* After dimension reduction, it is important to check the internal consistency of the training data (selected FPC’s scores) and the external variability between the training data from different states. Internal consistency refers to the similarity of observations within the same state, while external differences refer to the degree of dissimilarity between observations from different states. This check ensures that the classification methods can effectively distinguish between the different states.

**FIGURE 2:**
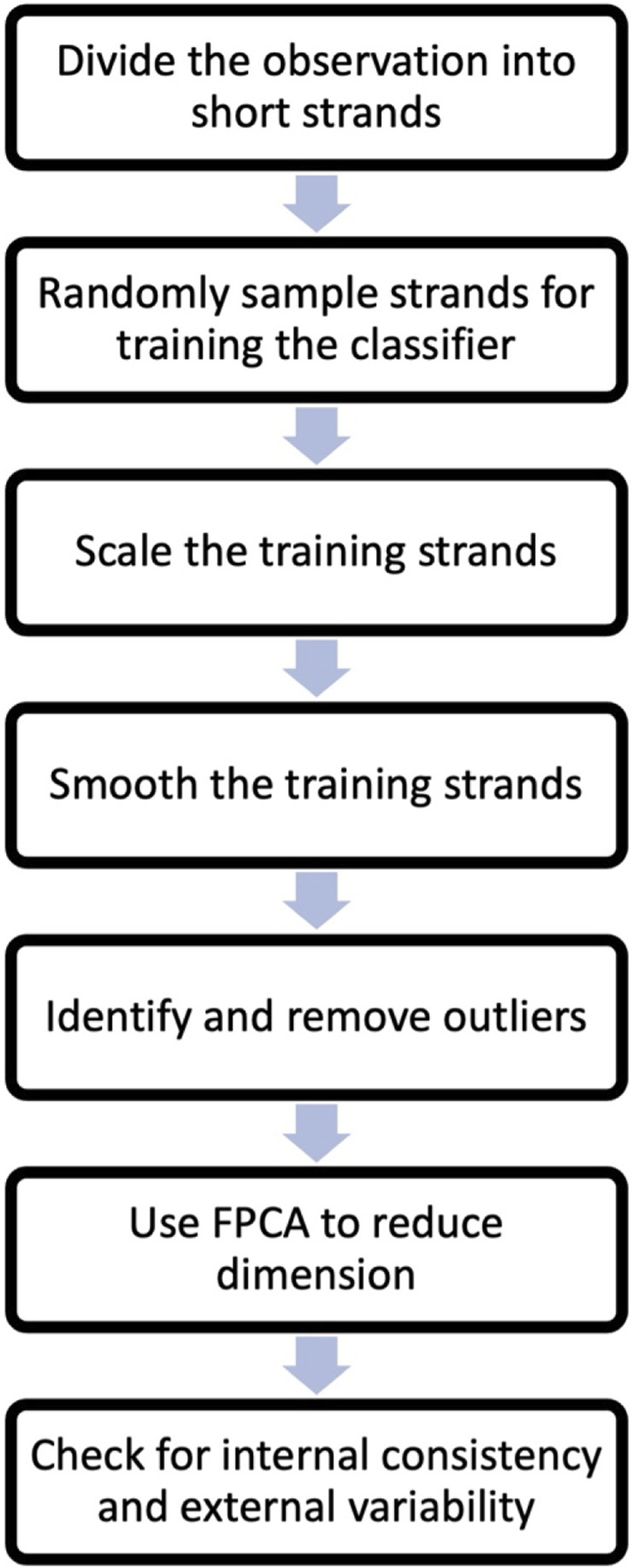
Flow chart of the steps leading to a trained classifier described in Section 3.1.

A flow chart based on the steps one to seven is included in Figure 2.

To assess internal consistency, a clustering algorithm can be employed to determine if the observations from a given state form *one* cluster. On the other hand, observations from different states should form *separate* clusters, indicating external variability. In this study, a model-based clustering method that fits a mixture of Gaussian distributions was used. Specifically, the Mclust 5 procedure in R was utilized (Fraley and Raftery, 2002; Scrucca et al., 2016; Scrucca, 2022). To evaluate the clustering models, the Bayesian Information Criterion (BIC) developed by Schwartz (Schwarz, 1978) was used as a criterion. BIC helps in selecting the most appropriate model by balancing model complexity and goodness of fit. This step ends the preprocessing stage of the AI analysis.

### 3.2 Training of the classifiers

Once the data preprocessing is completed, the next step is to train the classification/discrimination algorithm on the FPC scores. In this study, we used Model Based Clustering (MDA) and Random Forest (RF) methods. Specifically, we used the Gaussian Mixture Modeling for Model-Based Clustering, Classification, and Density Estimation R package Mclust (Scrucca et al., 2016; Scrucca, 2022). The Mclust package automatically selects the best classification or discrimination model based on the BIC. For the Random Forest work we used the R package randomForest (Breiman et al., 2022).

### 3.3 Classification of a new observation

To classify a new observation based on its nanomotion, the observation is divided into shorter strands, similar to the training data. A reasonable number of test strands (we recommend at least 30) are randomly sampled from the new observation for classification. The trained classifier is then applied to each test strand, and the result (assigned state) is recorded. The frequencies of the assigned states are computed, and the most frequent state is assigned to the entire nanomotion observation. Specifically, the steps of this method are:

Step 1: Divide the new observation, which can be, for example, 5 min long, into shorter strands, such as 5 s strands. In this case, you would have 60 strands of 5 s each.

Step 2: Apply the trained classifier to each of the 60 strands individually. Use the classifier to predict the state (virulent, avirulent, or dead) for every strand.

Step 3: Record the predicted state by the classifier for each strand. Keep track of the states predicted for all 60 strands.

Step 4: Compute the frequencies of all the predicted states, that is determine how many times each state was assigned by the classifier among the 60 strands.

Step 5: Assign the state with the highest frequency as the classification for the entire new nanomotion observation. If, for example, “virulent” was the most frequently predicted state among the 60 strands, assign the entire nanomotion observation as “virulent”.

**FIGURE 3:**
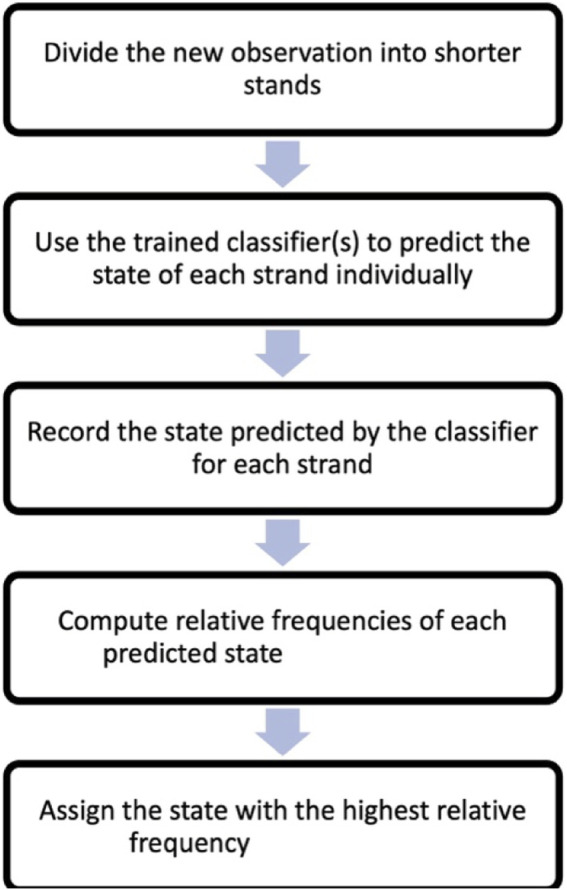
Flow chart of the steps leading to classification of a new observation following Section 3.3.

A flow chart based on the steps one to five above is included in Figure 3.

We used this classification method with MDA and RF classifiers. This approach considers the variability within the new observation and provides a quite robust classification method for the entire nanomotion observation. The accuracy of the classifier for individual 5 s strands and for the new observations is discussed in the section on the analysis of the B. pertussis nanomotion below.

Our approach resembles “voting” classification approaches (Cornelio et al. 2021, or Anton et al. 2023), where several classifiers are applied, and the predicted class is one predicted by the largest number of the classifiers. In turn, we divide the observation into many smaller chunks and apply the same classifier to each chunk. Then, the class predicted most often is the assigned class for the entire observation.

### 3.4 Analysis of the Bordetella pertussis nanomotion

In the B. pertussis example, the nanomotion samples consisted of 4 experimental replicates for each of the three states: virulent, avirulent, and dead. Each experimental replicate was a 5 min observation of the nanomotion.

**FIGURE 4:**
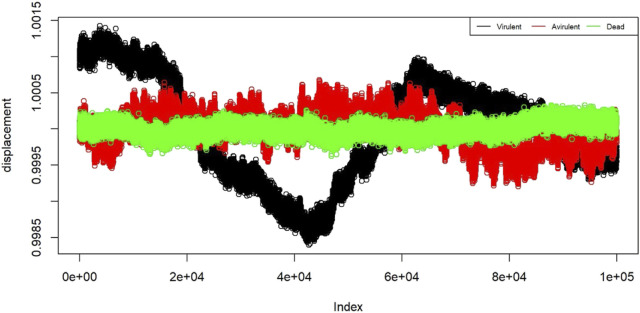
Displacement of the 12 example strands from the three states: virulent (black), avirulent (red), and dead (green). The horizontal axis shows the “index” which is the proxy for time. On the vertical axis we have displacement in the units of standard deviations.

To prepare the data for training the classification algorithm, each 5-min observation was divided into 60 strands of 5 s each. This resulted in a total of 240 strands per state, with 4 replicates for each state. From these strands, a random selection of 30 chunks per state was made, resulting in a total of 90 training chunks. These training chunks were further preprocessed and then used to train the classification algorithm. In Figure 4, a plot is shown with nanomotion data from the three states of the bacteria. The plot contains 12 randomly sampled strands from each state. The measurements are plotted in standard deviation units. Visualization of the displacement for different states helps to assess the internal similarity and external variability of the B. pertussis nanomotion.

### 3.5 Smoothing

To perform smoothing, a penalized 20-basis spline method was selected. This method is a standard choice for the data which is not periodic. The smoothed data was then subject to dimension reduction process.

### 3.6 Dimension reduction

Dimension reduction using FPCA was performed on the scaled and smoothed training data. We selected the top 6 FPCs which explained 98% of the total variability.

### 3.7 Outlier identification and removal

After obtaining the FPCs, outlier strands were identified and removed from the avirulent and dead samples using the technique described in the previous section. No outliers were identified among the virulent strands. Six outlier strands were removed from the avirulent sample, and nine outlier strands were removed from the dead sample. The remaining 30 strands from the virulent sample, 24 strands from the avirulent sample, and 21 strands from the dead sample were used for further analysis and training of the classifier.

### 3.8 Checking internal consistency and external differences

The clustering analysis using Mclust package confirmed one cluster per state for the virulent and avirulent strands, which indicated internal consistency within those states. The analysis showed two clusters for the dead strands, with the majority (86%) classified to the main cluster suggesting some variation within the dead state but still maintains reasonable internal consistency. There were three clusters identified for the data containing all training samples, which confirmed external variability needed for the discriminant analysis to work well.

### 3.9 Training of the classifiers

We used the Mclust (for MDA) and randomForest (for RF) R packages for training of the classifiers. The best model selected by Mclust was VEI-3 type.

### 3.10 Performance of the classifiers on the 5 s strands

The performance of the trained classifiers was evaluated by testing their accuracy on 150 randomly sampled 5 s strands. The strands were sampled from all 5 s strands which were not used in the training sample. The true state of each strand was noted, and the trained classification algorithms were used to predict their states. There were 55 virulent, 44 avirulent, and 51 dead strands among the 150 used for evaluation of the clarifiers. The resulting accuracy is summarized in Table 1 and Table 2.

**TABLE 1:** Accuracy of the MDA classifier on the 5 s strands.

		Predicted state		
		Virulent cells	Avirulent cells	Dead cells
True state	Virulent cell	55	0	0
	Avirulent cells	2	35	7
	Dead cells	0	4	47

**TABLE 2:** Accuracy of the RF classifier on the 5 s strands.

		Predicted state		
		Virulent cells	Avirulent cells	Dead cells
True state	Virulent cell	55	0	0
	Avirulent cells	1	31	12
	Dead cells	2	5	44

The overall error rate estimated using the 10-fold cross-validation method was found to be 26.84% for the MDA and 27.40% for the RF. This high overall error rates suggested that important information contained in the 5-min record was lost when reducing it to a 5 s strand.

### 3.11 Classification of a new 5-min observation

Classification of long (e.g., 5 min) observations has its own challenges. To build a classifier for such a long observation is prohibitive in computational time and requires very powerful machines. On the other hand, the classification of a 5-min observation based on one 5 s strand is not accurate, as we see above. In response to these considerations, a better classification method was developed specifically for new 5-min observations. This method was described in section 3.3. The assessment of the accuracy of this method was evaluated by constructing a test observation from a given state of bacteria. This test observation was created by randomly sampling sixty 5 s strands from the test set of strands. The trained classifier was then used to classify the state of each of the 60 individual strands. This process was repeated 200 times for each state, resulting in a total of 200 test classifications/predictions per state. The state with the highest frequency was predicted for a given 5-min test observation. We obtained perfect classification results using both classifiers (MDA and RF). That is all 200 of the 5-min long observations (random sets of sixty 5 s strands) were classified correctly for every state. The success of this method can be attributed to the statistical similarity among strands from the same state and enough variability among the strands from different states. The use of multiple strands from (possibly) different experimental replicates helped account for the internal variability within each state.

**FIGURE 5:**
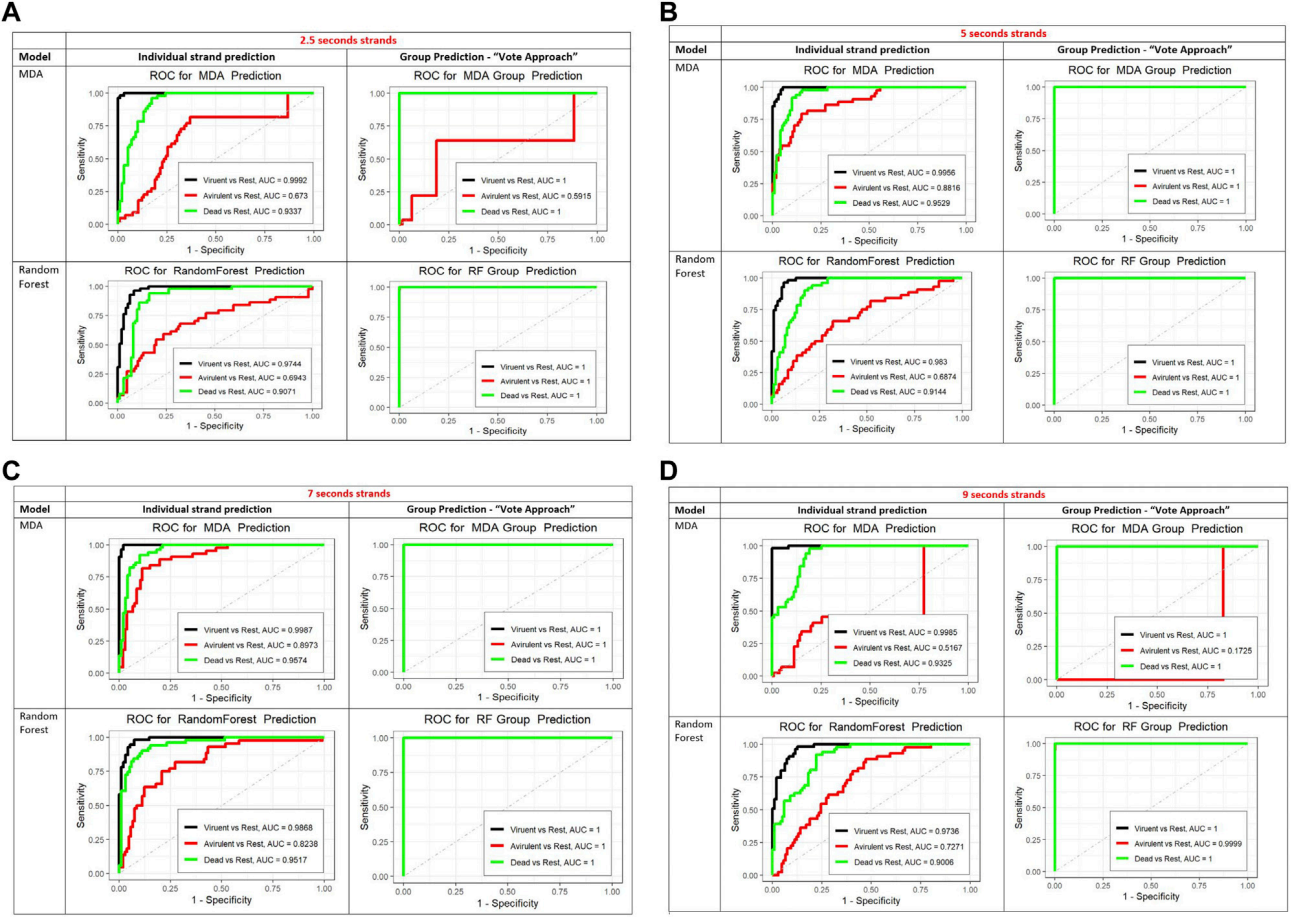
**(A)** The ROC curves for the prediction of individual 2.5 sec strands (left column) and for the vote method (group prediction approach, right column). For the MDA (top row) and RF (bottom row). In the group prediction column, top row the black curve (invisible) is identical to the green one (covers the black ROC curve). In the bottom row, group prediction column the black and red curves are identical to the green one and covered by it. The AUC in the legends of all ROC curves plots stands for Area Under the Curve. **(B)** The ROC curves for the prediction of individual 5 sec strands (left column) and for the vote method (group prediction approach, right column). For the MDA (top row) and RF (bottom row). In the group prediction column, in both rows the black and red curves are identical to the green one which covers them. The AUC in the legends of all ROC curves plots stands for Area Under the Curve. **(C)** The ROC curves for the prediction of individual 7 sec strands (left column) and for the vote method (group prediction approach, right column). For the MDA (top row) and RF (bottom row). In the group prediction column, in both rows the black and red curves are identical to the green one which covers them. The AUC in the legends of all ROC curves plots stands for Area Under the Curve. **(D)** The ROC curves for the prediction of individual 9 sec strands (left column) and for the vote method (group prediction approach, right column). For the MDA (top row) and RF (bottom row). In the group prediction column, bottom row (RF) the black and red curves are identical to the green one which covers them. In the group prediction MDA graph, the black curve is identical to the green one which covers it. The AUC in the legends of all ROC curves plots stands for Area Under the Curve.

To further assess the performance of the MDA and RF classifiers we created the ROC curves, presented in Figure 5B. For the ROC curves we looked at the three states separately, that is predicted one state against the other two states combined. Based on the areas under the ROC curves (AUC) both methods performed similarly for the dead and virulent states. MDA was slightly better than the RF for the avirulent state.

This high accuracy demonstrates the effectiveness of the classification algorithm and suggests that the nanomotion patterns captured by the AFM-based measurements contain significant information for distinguishing between different states of bacteria. Based on these findings, it is reasonable to conclude that this group classification method can be successfully applied to nanomotion classification of other states, bacteria, or organisms with similar degrees of internal consistency and external variability.

In addition, we performed a limited study of the performance of both classification methods for varying strand lengths: 2.5 s, 7 s, and 9 s. Figures 5A-D show the ROC curves for the prediction of individual strands and a new 5 min observation using strands of varying lengths (from 2.5 to 9 s), with MDA and RF methods. Both MDA and RF perform reasonably well. The RF algorithm performs better in predicting the class of the individual strands and in the vote method for the 2.5 and the 9 s strands (larger AUC). The difference is in the prediction of the avirulent state. The MDA predicts the class of the virulent and dead states for individual strands slightly better than the RF for all strands lengths. However, the RF is better in predicting the avirulent state with all strand lengths. Since the objective is classification of a new 5 min strand, the RF shows a more robust performance.

## 4 Discussion

Determining the optimal length of individual strands is an important aspect to consider. Shorter strands may capture specific dynamics or local patterns, while longer strands may provide a more comprehensive representation of the overall nanomotion. Finding a balance between the length of strands and computational feasibility is crucial. Similarly, determining the minimum number of strands required for accurate classification of a 5-min observation is an important consideration. It may depend on the complexity of the data and the level of variability between states. Further investigation and experimentation can help determine the optimal length and number of strands for effective classification.

The selection of the training set is another important aspect. While having more strands in the training set can improve the accuracy of the classifier, it also increases computational challenges. Finding the right balance between the number of training strands and computational efficiency is essential. It would be valuable to explore different scenarios and assess the impact of varying the number of training strands from each state on the performance of the classifier.

Additionally, considering the likelihood of each state (prior probability) occurring in the population when selecting the number of training strands can be beneficial. If certain states are more prevalent or have different levels of variability, adjusting the training set composition accordingly may lead to improved classification performance.

## 5 Conclusion

AFM-nanomotion is a very efficient tool to assess the sensitivity of bacteria against antibiotics. Up to now, live-dead classification was essentially relying on the nanomotion variance signal. More recently, it was demonstrated that the nanomotion signal also contains information on the metabolic state of the cells. However, classification of metabolic states is much more challenging than distinguishing life-dead states.

In this contribution, we propose a novel method for classifying bacterial virulence based on statistical analysis of nanomotion recordings. As a proof-of-concept, we could successfully classify living B pertussis bacteria in the virulent or avirulence phase, and dead state, based on their cellular nanomotion signal. Our method offers significant advantages over current approaches, as it is faster and more accurate.

With the fast-paced development of the artificial intelligence, computational methods based on our ideas should make the classification of nanomotion faster, more accurate and applicable to various biological or medical studies. In particular, ML/AI methods could be used in various studies of the nanomotion and in other applications.

## Data Availability

The raw data supporting the conclusion of this article will be made available by the authors, without undue reservation.

## References

[B1] Anton S. R. Martínez-Ojeda R. M. Hristu R. Stanciu G. A. Toma A. Banica C. K. (2023). Automated detection of corneal edema with deep learning-assisted second harmonic generation microscopy. IEEE JSTQE 29 (6), 1–10. 10.1109/JSTQE.2023.3258687

[B2] Binnig G. Quate C. F. Gerber C. (1886). Atomic force microscope. Phys. Rev. Lett. 56 (9), 930–933. 10.1103/physrevlett.56.930 10033323

[B3] Breiman L. (2001). Random forests. Mach. Learn. 45, 5–32. 10.1023/a:1010933404324

[B4] Breiman L. Cutler A. Liaw A. Wienerhttps M. (2022) e Breiman and Cutler's Random Forests for Classification and Regression Available at: https://www.stat.berkeley.edu/∼breiman/RandomForests/ .

[B5] Carbonetti N. H. Artamonova G. V. Andreasen C. Bushar N. (2005). Pertussis toxin and adenylate cyclase toxin provide a one-two punch for establishment of Bordetella pertussis infection of the respiratory tract. Infect. Immun. 73 (5), 2698–2703. 10.1128/IAI.73.5.2698-2703.2005 15845471 PMC1087369

[B6] Cornelio C. Donini M. Loreggia A. Pini M. S. Rossi F. (2021). Voting with random classifiers (VORACE): theoretical and experimental analysis. Aut. Agents Multi-Agent Syst. 35, 22. 10.1007/s10458-021-09504-y

[B7] Cotter P. A. Jones A. M. (2003). Phosphorelay control of virulence gene expression in Bordetella. Trends Microbiol. 11 (8), 367–373. 10.1016/s0966-842x(03)00156-2 12915094

[B8] Ester M. Kriegel H. P. Sander J. Xu X. (1996). “A density-based algorithm for discovering clusters in large spatial databases with noise,” in Proceedings of the Second International Conference on Knowledge Discovery and Data Mining 96. Portland, Oregon: AAAI Press, 226–231.

[B10] Fraley C. Raftery A. E. (2002). Model-based clustering, discriminant analysis, and density estimation. J. Am. Stat. Assoc. 458 (97), 611–631. 10.1198/016214502760047131

[B11] Hahsler M. Piekenbrock M. (2022). Dbscan: density-based spatial clustering of applications with noise (DBSCAN) and related algorithms. 11 R package version 1.1 Available at: https://CRAN.R-project.org/package=dbscan .

[B12] Hahsler M. Piekenbrock M. Doran D. (2019). Dbscan: fast density-based clustering with R. J. Stat. Softw. 91 (1), 1–30. 10.18637/jss.v091.i01

[B13] Hastie T. Tibshirani R. (1996). Discriminant analysis by Gaussian mixtures. J. R. Stat. Soc. Ser. B 58, 155–176. 10.1111/j.2517-6161.1996.tb02073.x

[B14] Kasas S. Ruggeri F. S. Benadiba C. Maillard C. Stupar P. Tournu H. (2015). Detecting nanoscale vibrations as signature of life. Proc. Natl. Acad. Sci. 112, 378–381. 10.1073/pnas.1415348112 25548177 PMC4299216

[B15] Kweku D. (2021). A combined functional data and mixture models approach for modeling and classification of nanomotions. master’s thesis. RENO (NV), University of Nevada Reno.

[B9] Li L. Deng J. Ma X. Zhou K. Meng Q. Yuan L. (2019). High Prevalence of Macrolide-Resistant Bordetella pertussis and ptxP1 Genotype, Mainland China. Emerg. Infect. Dis. 25 (12), 2205–2214. 10.3201/eid2512.181836 31742507 PMC6874251

[B16] Lissandrello C. Inci F. Francom M. Paul M. R. Demirci U. Ekinci K. L. (2014). Nanomechanical motion of *Escherichia coli* adhered to a surface. Appl. Phys. Lett. 105, 113701. 10.1063/1.4895132 25316924 PMC4187256

[B17] Longo G. Alonso-Sarduy L. Marques-Rio L. Bizzini A. Trampuz A. Notz J. (2013). Rapid detection of bacterial resistance to antibiotics using AFM cantilevers as nanomechanical sensors. Nat. Nanotech 8, 522–526. 10.1038/nnano.2013.120 23812189

[B18] Moon K. Bonocora R. P. Kim D. D. Chen Q. Wade J. T. Stibitz S. (2017). The BvgAS regulon of *Bordetella pertussis* . mBio 8 (5), e01526–17. 10.1128/mBio.01526-17 29018122 PMC5635692

[B19] Mustazzolu A. Venturelli L. Dinarelli S. Brown K. Floto R. A. Dietler G. (2019). A rapid unraveling of the activity and antibiotic susceptibility of mycobacteria. Antimicrob. Agents Chemother. 63 (3), e02194–18. 10.1128/AAC.02194-18 30602518 PMC6395931

[B20] Ng A. Y. Jordan M. I. Weiss Y. (2002). “On spectral clustering: analysis and an algorithm,” in Advances in Neural Information Processing Systems (NIPS), 849–856. 14 01.

[B21] Oh Y. J. Hinterdorfer P. (2018). Sensing the ultrastructure of bacterial surfaces and their molecular binding forces using AFM. Methods Mol. Biol. 1814, 363–372. 10.1007/978-1-4939-8591-3_21 29956243

[B22] Pleskova S. N. Lazarenko E. V. Bezrukov N. A. Bobyk S. Z. Boryakov A. V. Kriukov R. N. (2023b). Differences in bacteria nanomotion profiles and neutrophil nanomotion during phagocytosis. Front. Microbiol. 14, 1113353. 10.3389/fmicb.2023.1113353 37032906 PMC10076590

[B23] Pleskova S. N. Lazarenko E. V. Sudakova I. S. Kriukov R. N. Bezrukov N. A. (2023a). A new method for express detection of antibiotic resistance. Appl. Biochem. Microbiol. 59 (1), 73–78. 10.1134/s0003683823010076

[B24] Radonicic V. Yvanoff C. Villalba M. I. Devreese B. Kasas S. Willaert R. G. (2023). Single-cell optical nanomotion of Candida albicans in microwells for rapid antifungal susceptibility testing. Fermentation 9, 365. 10.3390/fermentation9040365

[B25] Relman D. Tuomanen E. Falkow S. Golenbock D. T. Saukkonen K. Wright S. D. (1990). Recognition of a bacterial adhesin by an integrin: macrophage CR3 (αMβ2,) binds filamentous hemagglutinin of Bordetella pertussis. Cell 61, 1375–1382. 10.1016/0092-8674(90)90701-f 2364431

[B26] Schwarz G. (1978). Estimating the dimension of a model. Ann. Statistics 6, 461–464. 10.1214/aos/1176344136

[B27] Scrucca L. (2022). A quick tour of mclust. Available at: https://cran.r-project.org/web/packages/mclust/vignettes/mclust.html (Accessed August 8, 2023).

[B28] Scrucca L. Fop M. Murphy T. B. Raftery A. E. (2016). Mclust 5: clustering, classification and density estimation using Gaussian finite mixture models. R J. 8 (1), 289–317. 10.32614/RJ-2016-021 27818791 PMC5096736

[B29] Stainer D. W. Scholte M. J. (1970). A simple chemically defined medium for the production of phase I Bordetella pertussis. J. Gen. Microbiol. 63, 211–220. 10.1099/00221287-63-2-211 4324651

[B30] Starodubtseva M. N. Irina A. Chelnokova I. A. Shkliarava N. M. Villalba M. I. Tapalski D. V. (2023). Modulation of the nanoscale motion rate of Candida albicans by X-rays. Front. Microbiol. 14, 1133027. 10.3389/fmicb.2023.1133027 37025638 PMC10070863

[B31] Stupar P. Opota O. Longo G. Prod'hom G. Dietler G. Greub G. (2017). Nanomechanical sensor applied to blood culture pellets: a fast approach to determine the antibiotic susceptibility against agents of bloodstream infections. Clin. Microbiol. Infect. 23, 400–405. 10.1016/j.cmi.2016.12.028 28062319

[B32] Stupar P. Podolski-Renic A. Villalba M. I. Dragoj M. Stojanov S. J. Pešic M. (2021). Nano-Motion analysis for rapid and label free assessing of cancer cell sensitivity to chemotherapeutics. Medicina 57, 446. 10.3390/medicina57050446 34064439 PMC8147836

[B33] Vadillo-Rodríguez V. Busscher H. J. Norde W. de Vries J. Dijkstra R. J. B. Stokroos I. (2004). Comparison of atomic force microscopy interaction forces between bacteria and silicon nitride substrata for three commonly used immobilization methods. Environ. Microbiol. 70, 5441–5446. 10.1128/AEM.70.9.5441-5446.2004 PMC520872 15345431

[B34] Venturelli L. Harrold Z. R. Murray A. E. Villalba M. I. Lundin E. M. Dietler G. (2021). Nanomechanical bio-sensing for fast and reliable detection of viability and susceptibility of microorganisms. Sensors Actuators B Chem. 348, 130650. 10.1016/j.snb.2021.130650

[B35] Venturelli L. Kohler A.-C. Stupar P. Villalba M. I. Kalauzi A. Radotic K. (2020). A perspective view on the nanomotion detection of living organisms and its features. J. Mol. Recognit. 33, e2849. 10.1002/jmr.2849 32227521

[B36] Villalba M. I. Rosetti E. Bonvallant A. Kasas S. Radonicic V. Willaert R. G. (2023). Simple optical nanomotion method for single-bacterium viability and antibiotic response testing. PNAS 120 (18), e2221284120. 10.1073/pnas.2221284120 37094120 PMC10160964

[B37] Villalba M. I. Stupar P. Chomicki W. Bertacchi M. Dietler G. Arnal L. (2018). Nanomotion detection method for testing antibiotic resistance and susceptibility of slow-growing bacteria. Small 14, 1702671. 10.1002/smll.201702671 29205867

[B38] Villalba M. I. Venturelli L. Arnal L. Masson C. Dietler G. Vela M. E. (2022). Effect of antibiotics on mechanical properties of Bordetella pertussis examined by atomic force microscopy. Micron 155 (103229), 103229. ISSN 0968-4328. 10.1016/j.micron.2022.103229 35149252

[B39] World Health Organization (2023). Antimicrobial resistance. Available at: https://www.who.int/news-room/fact-sheets/detail/antibiotic-resistance (Accessed August 8, 2023).

[B40] Wu S. Liu X. Zhou X. Liang X. M. Gao D. Liu H. (2016). Quantification of cell viability and rapid screening anti-cancer drug utilizing nanomechanical fluctuation. Biosens. Bioelectron. 77, 164–173. 10.1016/j.bios.2015.09.024 26406457

